# Analysis of Mechanical Properties and Mechanical Anisotropy in Canine Bone Tissues of Various Ages

**DOI:** 10.1155/2019/3503152

**Published:** 2019-06-20

**Authors:** Changqi Luo, Junyi Liao, Zhenglin Zhu, Xiaoyu Wang, Xiao Lin, Wei Huang

**Affiliations:** Department of Orthopedic Surgery, The First Affiliated Hospital of Chongqing Medical University, No. 1, Youyi Road, Yuanjiagang, Yuzhong District, Chongqing 400016, China

## Abstract

The effect of age on mechanical behavior and microstructure anisotropy of bone is often ignored by researchers engaged in the study of biomechanics. The objective of our study was to determine the variations in mechanical properties of canine femoral cortical bone with age and the mechanical anisotropy between the longitudinal and transverse directions. Twelve beagles divided into three age groups (6, 12, and 36 months) were sacrificed and all femurs were extracted. The longitudinal and transverse samples of cortical bone were harvested from three regions of diaphysis (proximal, central, and distal). A nanoindentation technique was used for simultaneously measuring force and displacement of a diamond tip pressed 2000nm into the hydrated bone tissue. An elastic modulus was calculated from the unloading curve with an assumed Poisson ratio of 0.3, while hardness was defined as the maximal force divided by the corresponding contact area. The mechanical properties of cortical bone were determined from 852 indents on two orthogonal cross-sectional surfaces. Mean elastic modulus ranged from 7.56±0.32 GPa up to 21.56±2.35 GPa, while mean hardness ranged from 0.28±0.057 GPa up to 0.84±0.072 GPa. Mechanical properties of canine femoral cortical bone tended to increase with age, but the magnitudes of these increase for each region might be different. The longitudinal mechanical properties were significantly higher than that of transverse direction (*P*<0.01). A significant anisotropy was found in the mechanical properties while there was no significant correlation between the two orthogonal directions in each age group (*r*^2^<0.3). Beyond that, the longitudinal mechanical properties of the distal region in each age group were lower than the proximal and central regions. Hence, mechanical properties in nanostructure of bone tissue must differ mainly among age, sample direction, anatomical sites, and individuals. These results may help a number of researchers develop more accurate constitutive micromechanics models of bone tissue in future studies.

## 1. Introduction

The bone material is an inhomogeneous multilayer composite structure and includes a series of components such as osteonal bone, interstitial bone, laminar bone, and trabecular bone in different regions. The mechanical properties of bone tissue are determined by composition as well as structural, microstructural, and nanostructural organization [1]. Elastic modulus and hardness are typical indicators for evaluating the mechanical properties of bone tissue. Many researchers have gradually turned from the macroscopic mechanics of bones to the micromechanical level in recent years, and several studies indicate that the age-related variations may cause the changes of cortical bone in the microstructural levels and therefore influence its mechanical behavior [2–6]. However, it is not well known whether the mechanism is closely related to the age-related changes in the mechanical behavior of the bone itself. In addition, the existing models always focus on the single longitudinal properties and neglect the bone anisotropy which is not conducive to the analysis of the effect of microstructural changes on the overall mechanical properties of bone [1, 7, 8]. Therefore, fully understanding the effect of age on the mechanical behavior of bone and bone microstructure anisotropy is of great importance to explore multiscale constitutive micromechanics models.

The nanoindentation technology proposed by Oliver and Pharr has become a new technology for studying the micromechanical properties of materials [9]. Unlike the more conventional microhardness techniques, nanoindentation provides both elastic modulus and hardness estimates for a material and can be used to target specifically various bone tissue structures at a microscopic level [10, 11]. The mechanical properties of the material, such as load-displacement curves, elastic modulus, hardness, fracture toughness, strain hardening effect, viscoelasticity, or creep behavior, can be obtained by a needle-like indenter pressed into the surface of the sample [12–14].

Therefore, the objective of this work was to determine the variations in mechanical properties of canine femoral cortical bone with age and the mechanical anisotropy between the longitudinal and transverse directions under hydrated conditions by using nanoindentation technology. In separate studies, some researchers reported the mechanical properties of bone tissue in porcine, bovine, and mouse models, respectively. However, they did not comprehensively analyze the effects of age and region on mechanical properties and mechanical anisotropy [15, 16]. Canine bone was chosen because its tissue structure, systematic function, and fracture healing process were similar to humans and there was no published data on the mechanical properties of such bone to our knowledge [17, 18]. Bone samples from three different age groups (6, 12, and 36 months), representing developmental bone, ranging from young to mature animal models, were selected for testing so that young, developing, and adult bones can be studied. The stages of bone development correspond to human levels of 8, 18, and 30 years [19].

## 2. Materials and Methods

### 2.1. Sample Preparation

Ethical approval for the study was obtained through the Ethics Committee of the First Affiliated Hospital of Chongqing Medical University following Institutional Animal Care (No. 2019-025). Twelve beagles divided into three age groups (6, 12, and 36 months) were sacrificed and all femurs were extracted. The longitudinal and transverse samples obtained from femoral cortical bone were harvested from three regions of diaphysis (proximal, central, and distal). The proximal, central, and distal regions corresponded to an area of 2-3cm, 5-6cm, and 8-9cm below the femoral lesser trochanter, respectively.

**Figure 1:**
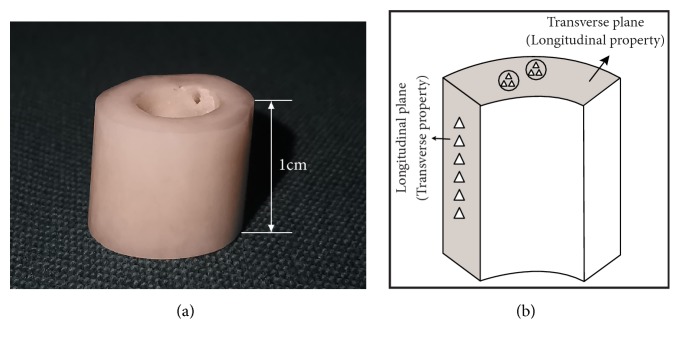
(a) A 1cm high annular cylinder extracted from femoral cortical bone of diaphysis (proximal, central, and distal). (b) Location of the indents on the longitudinal and transverse plane. The white triangles indicate six indents in the shaded area.

**Figure 2:**
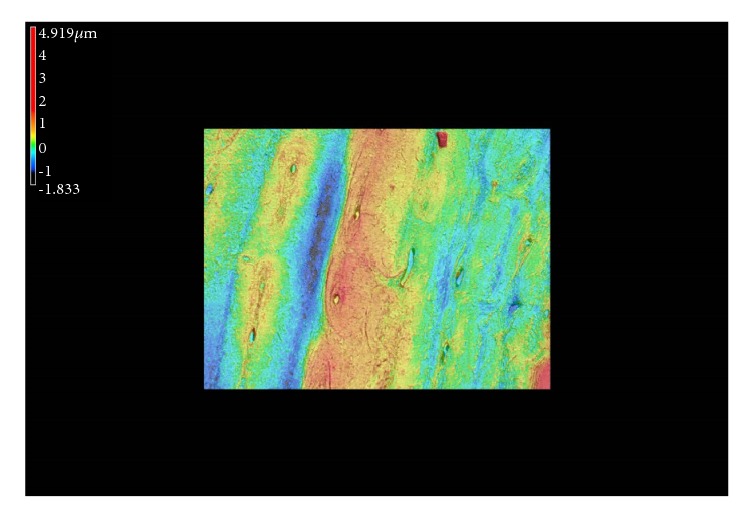
LSCM image of the surface roughness after sample processing.

The samples had their soft tissue removed using a soft water jet followed by an ultrasonic bath. Then, they were stored in a refrigerator at -20°C immersed in physiological buffer and naturally thawed at 4°C before segmentation. Bone samples were sectioned into 3mm slices (approximately a quarter of the circumference, from the posterior and lateral aspects of the three regions) along a transverse plane from the 1cm high annular cylinder (Figure 1(a)). Cutting process was under constant deionized water irrigation with saw blade (SATA, 10in, USA) and a precision diamond band saw (IsoMet, Buehler, 500r/s, USA). The purpose of an irrigation procedure was to reduce the adverse effects of local exotherm and minimize minerals remaining on the surface of the samples. All samples were subjected to exactly the same cleaning and mounting protocol simultaneously and embedded in polymethyl methacrylate (PMMA) at room temperature (23°C). After rehydration, the embedded samples were afterwards ground step by step with silicon carbide abrasive paper of 600 grift to 2000 grit size (600, 800, 1200, and 2000 grit) and metallographically polished on 0.05*μ*m alumina powder microcloths until the surface was smooth as a mirror. Finally, the debris on the surface of the sample was washed with an ultrasonic cleaner and rinsed again with deionized water for 3 minutes. Each sample was imaged with laser confocal microscope (LSCM) to quantify surface roughness (Figure 2).

### 2.2. Nanoindentation Measurements

The samples were taken out of the refrigerator and thawed naturally at room temperature. All experiments were conducted using a nanoindenter (Micro Materials, TM600, UK) under condition that the laboratory was kept at a normal temperature of 23°C and a humidity of 60% to 70%. The system had force and displacement resolutions of 0.3*μ*N and 0.16nm, respectively. A sensitive computer was used for simultaneously measuring force and displacement of a sharp diamond Berkovich tip pressed 2000nm into the hydrated bone tissue. The tests imposed a maximum load of 50mN with a loading rate of 0.4mN/s and unloading rate of 0.9mN/s. And then the indenter was slowly driven to the surface at a rate of 10nm/s until surface contact was detected by changes in the load and displacement signals. A constant load was held for 10s after removing 85% of the maximal load to eliminate the viscoelastic effects [20, 21].

**Figure 3:**
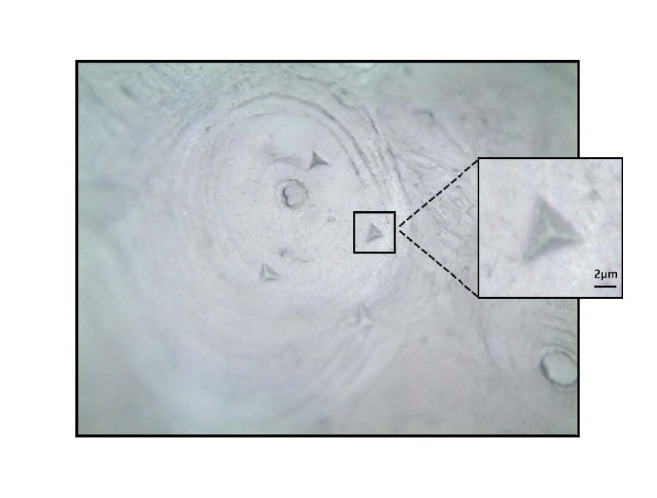
Microscopic image shows the shape of the typical triangular pyramidal indentations impression (×200).

The longitudinal and transverse data was obtained from two orthogonal planes, respectively, as shown in Figure 1(b). Six sites were examined in each target area and each site was maintained at the same distance [22]. The indent areas were selected in the middle half of the cortical bone on the longitudinal plane while the indents were located in osteons on the transverse plane to ensure the authenticity of the data. The typical pyramidal indentation impressions were observed in Figure 3 using a metalloscope (Olympus BX60, Japan).

**Figure 4:**
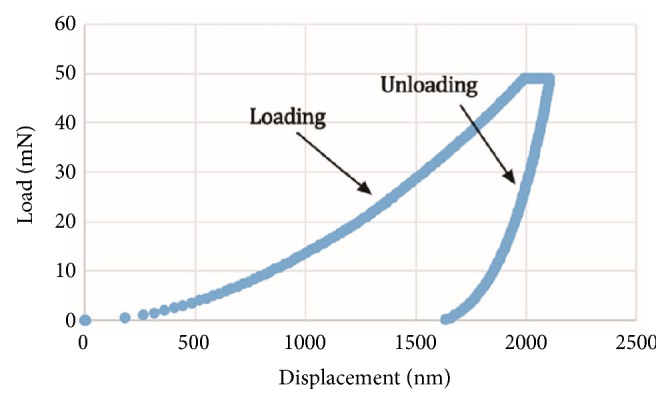
A typical force-displacement curve from a nanoindentation test. The elastic modulus and hardness are determined from the data during one cycle of loading and unloading. Hardness is the peak load at the projected area, while the elastic modulus is computed from the initial slope of unloading segment.

Elastic modulus and hardness of the bone tissue were calculated from the force-displacement curves. A typical indentation test resulted in a loading and unloading curve as shown in Figure 4. The mathematical solution was adapted for the nanoindenter by Oliver and Pharr and an elastic modulus was calculated from the unloading curve with an assumed Poisson ratio of 0.3 [9]. The initial slope of the unloading curve is the contact stiffness of the indenter (*S*) while the unloading stiffness* S *is related to the elastic modulus *E*_r_ by

(1) $$\begin{array}{c} S=\beta \frac{2}{\sqrt{\pi }}E_{r}\sqrt{A} \end{array}$$

where* A* is the projected area of contact and *β* is an empirical shape factor. For a Berkovich diamond indent, the shape factor is 1.034. The hardness (*H*) is defined as the peak indentation load (*P*max) divided by the projected area of the contact impression (*A*).

(2) $$\begin{array}{c} H=\frac{P_{max}}{A} \end{array}$$

### 2.3. Statistical Analyses

Statistical analysis was performed using the software SPSS version 22.0 (IBM Corporation, Armonk, NY). Quantitative data was expressed as mean value ± standard deviation. A one-way ANOVA test was performed between the three age groups on elastic modulus of indentations in the central region. Two-sample* t*-test was performed to compare the data points for each age group in two orthogonal directions. The same statistical analysis was also used for hardness in the corresponding regions. In addition, a paired sample* t*-test was performed between the data obtained from left femurs and right femurs. According to a Turkey test, the results of three regions were further marked in different statistical groups with p=0.05. The Pearson correlation coefficients were calculated to investigate possible correlations between the two orthogonal directions for elastic modulus and hardness. The coefficient* r* indicates the degree of linear correlation between the two variables. For all analyses, a 2-tailed value of* P*<0.05 is defined as statistically significant.

## 3. Results

### 3.1. Elastic Modulus and Hardness

Material surface roughness was one of the factors affecting the results of nanoindentation and all samples were consistent with the standard for the test [23]. About 12 sets of elastic modulus and hardness values may not be obtained due to water intrusion on the surface of the tissue. In total, 852 indents were made for statistical analyses. The drifting effect of the displacement transducer was found to be negligible in the calculation of the mechanical properties as the difference of the values between corrected and uncorrected data was less than 2%.

**Figure 5:**
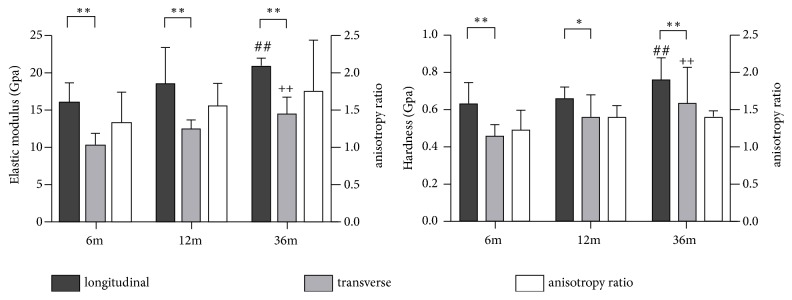
The distributions of mechanical properties in the central region. ^∗∗^*p *< 0.01 when compared to transverse results in the same age group; ^∗^*p *< 0.05 when compared to transverse results in the same age group; ^##^*p *< 0.05 when compared to longitudinal results in 6-month and 12-month group; ^++^*p *< 0.05 when compared to transverse results in 6-month and 12-month group.

**Figure 6:**
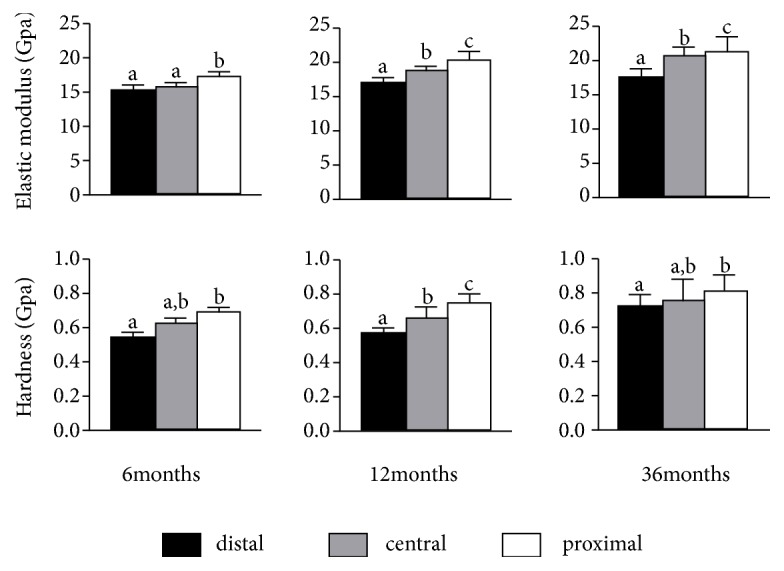
Longitudinal elastic modulus and hardness of three age groups as a function of bone region. a, b, c Any two groups that have diverse letters are statistically different at a significance level of p<0.05.

The average elastic modulus of the six indents ranged from 7.56±0.32 GPa (transverse direction sample in the central region from 6 months group) up to 21.56±2.35 GPa (longitudinal direction sample in the proximal region from the 36-month group), while the average hardness of the six indents ranged from 0.28±0.057 GPa (transverse direction sample in the proximal region from 6 months group) up to 0.84±0.072 GPa (longitudinal direction sample in the proximal region from the 36-month group). The statistical difference of the average elastic modulus and hardness between left and right femurs in the same directions was not detected. As a result, the distributions of mechanical properties for the central samples showed that the elastic modulus and hardness in 36-month samples proved to be statistically higher than the 6-month and 12-month samples (P<0.05) (Figure 5). The anisotropy ratio was defined as the ratio between the two orthogonal values of the mechanical properties [16]. Moreover, the longitudinal mechanical properties of canine femoral cortical bone were significantly higher than those of transverse direction according to the* t*-test result (P<0.01) except for the hardness in 12-month samples (P<0.05) (Figure 5). The statistical data of anisotropy ratios for the elastic modulus and hardness was demonstrated in Figure 5. Similar trends were also observed in other two regions. As shown in Figure 6, the longitudinal mechanical properties of the distal region in each age group were statistically lower than the proximal and central regions (*P*<0.05). The exception was the elastic modulus between the distal and central regions in the 6-month group.

### 3.2. Relationship of Mechanical Properties between Two Directions

**Figure 7:**
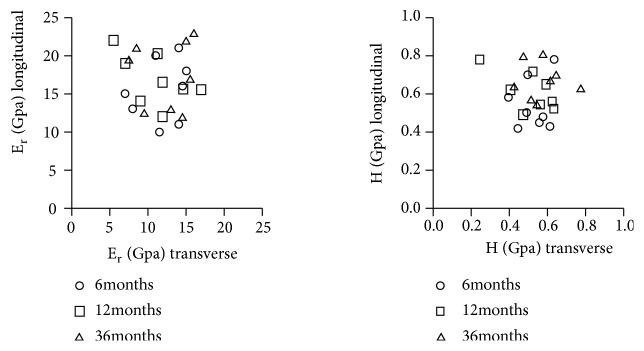
Scatter diagrams of the elastic modulus and hardness found in the two orthogonal directions in the central region. Each element in the plot represents an age group (6 months, 12 months, or 36 months) (24 femurs in total). No correlation was observed for two mechanical parameters.

The longitudinal and transverse data of elastic modulus and hardness was obtained from the transverse and longitudinal plane, respectively. A simple tool measurement was used to make sure the two test surfaces were perpendicular to each other. We failed to observe a correlation between two orthogonal directions for two mechanical parameters from our scatter diagrams (r^2^<0.3, Figure 7).

## 4. Discussion and Conclusion

The first attempt to quantify the mechanical properties of bone microstructure was a microhardness test with an indent size of 50*μ*m and a weight of 100g [24]. In particular, the hardness values between 0.049 and 0.579 GPa for fresh human bone were obtained from Weaver's extensive study. Nanoindentation technique has been widely used in the mechanical study of hard and soft tissue materials in recent years. It provides a means by which the inherent mechanical properties can be directly obtained since most of the microstructure features in bone are a few microns or more in dimension [25].

Previous studies have also measured the elastic modulus and hardness of porcine, bovine, and mouse bone tissue. Feng [15] et al. reported that both the elastic modulus and hardness increased with age using samples of porcine femoral cortical bone, while the possible differences in the mechanical properties between different regions and different directions in the same region were ignored. Further analysis revealed that the developmental effect was not uniform through the bone microstructure and could be attributed to different levels of mineralization or differences in collagen fibril orientation. A simple measurement of the mechanical properties of the mouse femoral cortical bone using nanoindentation was achieved by Tang [26] et al. The average elastic modulus was 42.11±11.72 GPa and hardness 1.85±0.13 GPa for sham group and 31.35±1.81 GPa and 1.33±0.15 GPa, respectively, for ovariectomized group. The major problem was also the limitation of a single region and direction. Some insight into this problem can be gained from the work of Casanova [16] et al. A significant anisotropy in the mechanical properties and variations between different regions of bone tissue were observed from that work. However, no analysis of the age-related effects on the mechanical behavior of bone tissue was performed. To the best of our knowledge, we are the first to use canine micromechanical models to analyze all those factors.

In the present study, we have observed that the elastic modulus and hardness of canine femoral cortical bone tend to increase with age, but the magnitudes of this increase for each region may be different. It should be remembered that knowledge of the changes that exist in bone with age and between individuals is essential to the understanding of how and why these changes come about. Our results partly address the effect of age on mechanical properties of bone tissue. The osteocyte proliferation and differentiation, cytokines, and hormone levels are involved in the development of bone tissue. These factors affect the remodeling process of bone microstructure with the increase of age, thus leading to the variation in mechanical properties. The variation may be also related to mineralization levels, collagen fiber orientation, and differences in collagen cross-linking within the three regions during the development of bone tissue [3, 27].

It is interesting to examine the anisotropy of canine femur in mechanical properties in the longitudinal and transverse directions. Studies on other types of bone tissue have found that the anisotropic ratio of elastic modulus at the microstructural level ranges from 1.5 to 2.0, and the values in hardness vary from 1.1 to 1.3 [28, 29]. Our observations are similar to the results of that literature. The needle-shaped hydroxyapatite crystals and collagen fibers are mainly arranged in the longitudinal direction according to the main direction of force. On the other hand, a few of them are arranged in the circumferential direction as a role of bridging. The main function is to associate and constrain the longitudinal fibers to keep them stable under the action of compression and bending. Given this perspective, it is reasonable to explain the fact that the longitudinal mechanical properties of canine femoral cortical bone were significantly higher than those of transverse direction. However, we failed to observe a correlation between two orthogonal directions for two mechanical parameters in the same region. The finding contradicts the original assumption by Carter that the mechanical properties of bone tissue in one direction can be predicted from the other direction perpendicular to it [30].

The amount of collagen fiber varies from one region to another. And the distribution of collagen fiber orientation may also influence the interpretation of the elastic modulus and hardness of the cortical bone. The difference in mechanical properties found between anatomical sites may be related to turnover rate and osteon type. A higher turnover rate reduces the average age of the osteons, thus reducing mineralization and associated mechanical properties. Moreover, Ascenzi et al. have shown that the femoral neck may comprise different distributions of osteon type (characterized by the predominant collagen fiber orientation) compared to the diaphysis, which may also result in distinct average mechanical properties [31]. The same phenomenon may be present in different regions of the diaphysis. In combination with the results of Ascenzi et al., the present analysis reveals the gradient variation of the longitudinal mechanical properties along the longitudinal axis in each age group. In addition to the above analyses, another possible reason could be explained by the fact that the geometrical morphology of femurs changes from proximal to distal region, resulting in malalignment of collagen fibers and indents on the longitudinal plane. However, indents on the longitudinal plane may not be affected in the same age group. The variation is also in line with the principle of mechanical transmission under physiological conditions.

In conclusion, mechanical properties in nanostructure of bone tissue must differ mainly among age, sample direction, anatomical sites, and individuals. The sophisticated constitutive mechanical models should include all these factors. The findings would be valuable for providing theoretical basis for future study on micromechanics using canine animal models and could also provide useful information in understanding the complex interactions of biological and cellular mechanisms for ostial disorders.

The major limitation of this study is the difficulty of trabecular bone measurement, which is unable to provide complete information about mechanical properties of cortical and trabecular bone. The comprehensive analysis of bone's microstructure (laminar bone, interstitial bone, and osteons) and a larger sample size are also needed for future studies.

## Data Availability

The data used to support the findings of this study are included within the article.

## References

[1] Zysset P. K., Guo X. E., Hoffler C. E., Moore K. E., Goldstein S. A. (1999). Elastic modulus and hardness of cortical and trabecular bone lamellae measured by nanoindentation in the human femur. *Journal of Biomechanics*.

[2] Fan Z., Swadener J. G., Rho J. Y., Roy M. E., Pharr G. M. (2002). Anisotropic properties of human tibial cortical bone as measured by nanoindentation. *Journal of Orthopaedic Research*.

[3] Oyen M. L. (2006). Nanoindentation hardness of mineralized tissues. *Journal of Biomechanics*.

[4] Rho J.-Y., Roy M. E., Tsui T. Y., Pharr G. M. (1999). Elastic properties of microstructural components of human bone tissue as measured by nanoindentation. *Journal of Biomedical Materials Research Part B: Applied Biomaterials*.

[5] Rho J.-Y., Tsui T. Y., Pharr G. M. (1997). Elastic properties of human cortical and trabecular lamellar bone measured by nanoindentation. *Biomaterials*.

[6] Rho J. Y., Zioupos P., Currey J. D., Pharr G. M. (2002). Microstructural elasticity and regional heterogeneity in human femoral bone of various ages examined by nano-indentation. *Journal of Biomechanics*.

[7] Zebaze R. M. D., Jones A. C., Pandy M. G., Knackstedt M. A., Seeman E. (2011). Differences in the degree of bone tissue mineralization account for little of the differences in tissue elastic properties. *Bone*.

[8] Zhang Z., Jia H., Sun J., Tong J. (2016). Nanoindentation investigation of the stress exponent for the creep of dung beetle (Copris ochus Motschulsky) cuticle. *Bioengineered*.

[9] Oliver W. C., Pharr G. M. (1992). Improved technique for determining hardness and elastic modulus using load and displacement sensing indentation experiments. *Journal of Materials Research*.

[10] Doyran B., Tong W., Li Q. (2017). Nanoindentation modulus of murine cartilage: a sensitive indicator of the initiation and progression of post-traumatic osteoarthritis. *Osteoarthritis and Cartilage*.

[11] Hoffler C. E., Moore K. E., Kozloff K., Zysset P. K., Brown M. B., Goldstein S. A. (2000). Heterogeneity of bone lamellar-level elastic moduli. *Bone*.

[12] Swadener J. G., Rho J.-Y., Pharr G. M. (2001). Effect of anisotropy on elastic moduli measured by nanoindentation in human tibial cortical bone. *Journal of Biomedical Materials Research Part B: Applied Biomaterials*.

[13] Vashishth D., Tanner K. E., Bonfield W. (2001). Fatigue of cortical bone under combined axial-torsional loading. *Journal of Orthopaedic Research*.

[14] O'Brien F. J., Hardiman D. A., Hazenberg J. G. (2005). The behaviour of microcracks in compact bone. *European Journal of Morphology*.

[15] Feng L., Chittenden M., Schirer J., Dickinson M., Jasiuk I. (2012). Mechanical properties of porcine femoral cortical bone measured by nanoindentation. *Journal of Biomechanics*.

[16] Casanova M., Balmelli A., Carnelli D., Courty D., Schneider P., Müller R. (2017). Nanoindentation analysis of the micromechanical anisotropy in mouse cortical bone. *Royal Society Open Science*.

[17] Rhinelander F. W., Phillips R. S., Steel W. M., Beer J. C. (1968). Microangiography in bone healing. II. Displaced closed fractures. *The Journal of Bone & Joint Surgery*.

[18] Melissa P., Sean D., Susan L. (2009). Canine tumor cross-species genomics uncovers targets linked to osteosarcoma progression. *BMC Genomics*.

[19] Debra M. E., Liisa D. C., Delbert G. C. (2007). *Comparative Age of Dogs and Humans*.

[20] Pathak S., Gregory Swadener J., Kalidindi S. R., Courtland H.-W., Jepsen K. J., Goldman H. M. (2011). Measuring the dynamic mechanical response of hydrated mouse bone by nanoindentation. *Journal of the Mechanical Behavior of Biomedical Materials*.

[21] Lucca D. A., Herrmann K., Klopfstein M. J. (2010). Nanoindentation: Measuring methods and applications. *CIRP Annals - Manufacturing Technology*.

[22] Lucchini R., Carnelli D., Ponzoni M., Bertarelli E., Gastaldi D., Vena P. (2011). Role of damage mechanics in nanoindentation of lamellar bone at multiple sizes: Experiments and numerical modeling. *Journal of the Mechanical Behavior of Biomedical Materials*.

[23] Ho S. P., Goodis H., Balooch M., Nonomura G., Marshall S. J., Marshall G. (2004). The effect of sample preparation technique on determination of structure and nanomechanical properties of human cementum hard tissue. *Biomaterials*.

[24] Weaver J. K. (1966). The microscopic hardness of bone. *The Journal of Bone & Joint Surgery*.

[25] Silva M. J., Brodt M. D., Fan Z. F., Rho J.-Y. (2004). Nanoindentation and whole-bone bending estimates of material properties in bones from the senescence accelerated mouse SAMP6. *Journal of Biomechanics*.

[26] Tang B., Ngan A. H. W., Lu W. W. (2007). An improved method for the measurement of mechanical properties of bone by nanoindentation. *Journal of Materials Science: Materials in Medicine*.

[27] Franzoso G., Zysset P. K. (2009). Elastic anisotropy of human cortical bone secondary osteons measured by nanoindentation. *Journal of Biomechanical Engineering-Transactions of the ASME*.

[28] Carnelli D., Lucchini R., Ponzoni M., Contro R., Vena P. (2011). Nanoindentation testing and finite element simulations of cortical bone allowing for anisotropic elastic and inelastic mechanical response. *Journal of Biomechanics*.

[29] Reisinger A. G., Pahr D. H., Zysset P. K. (2011). Elastic anisotropy of bone lamellae as a function of fibril orientation pattern. *Biomechanics and Modeling in Mechanobiology*.

[30] Carter D. R., Hayes W. C. (1977). The compressive behaviour of bone as a two phase porous structure. *The Journal of Bone & Joint Surgery*.

[31] Ascenzi A., Baschieri P., Benvenuti A. (1994). The torsional properties of single selected osteons. *Journal of Biomechanics*.

